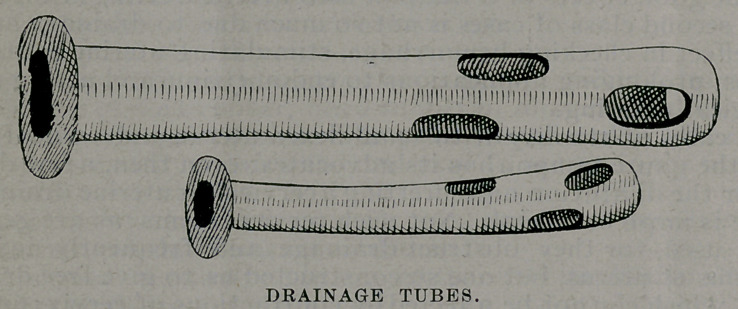# Uterine Drainage as a Factor in the Prevention and Relief of Pelvic Inflammation

**Published:** 1897-02

**Authors:** R. R. Kime

**Affiliations:** Atlanta, Ga.


### UTERINE DRAINAGE AS A FACTOR IN THE PREVENTION AND RELIEF OF PELVIC INFLAMMATION.

BY R. R. KIME, M. D., ATLANTA, GA.

He who prevents or relieves disease without removal of useful organs is a greater benefactor to mankind than the one who sacrifices such organs because of his inability to restore them to their normal functions.

When we consider how frequently removal of the tubes and ovaries, even the uterus itself, fails to relieve the patient to the extent desired, that she thus encounters the risk of death, intestinal adhesions, hernia, various varieties of fistulæ, ultimately lessened sexual power, sterility and the various mental and nervous phenomena that accompany such operations it is time we study preventive gynecology as well as preventive medicine.

It has been estimated that 50 to 60 per cent. of diseased tubes and ovaries requiring removal are due to infection after abortion or labor, and that 25 per cent. are due to gonorrheal infection.

If these statements approximate the truth then I need not apologize for presenting so trite a subject as uterine drainage before such an assemblage as this, especially when I feel that it is one of the most important factors in the prevention and treatment of pelvic inflammation.

When and how to drain in abdominal work has been a vexed question and has called forth much discussion in the last few years.

Vaginal work is fast solving the question and with the aid of the microscope, it is hoped more definite rules will soon be formulated. Vaginal work has also aided materially in the solution of uterine drainage.

We have two varieties of uterine drainage which may be utilized to a greater or less extent in three general classes of cases:

1. In endometritis, metritis, sterility.
2. Incomplete abortions with infection.
3. Puerperal infection.

Capillary drainage is secured by gauze or wicking, tubular, by the use of soft rubber, hard rubber or aluminum drainage tubes properly constructed. The *gauze tampon* and *cervical stems or plugs* as usually constructed and used do not drain the uterus efficiently.

Gauze or wicking when properly used drains off liquids only, not germs, cellular elements or debris.

When used as a tampon it acts, as its name implies, to check, obstruct or fill up.

It is packed in the abdominal cavity to check hemorrhage, hence obstructs, especially so in the uterine cavity.

We have important factors that interfere with gauze drainage that are inherent to the uterus itself.

1. The uterine cavity is increased or diminished by contraction or reaction of muscular tissue in the uterine wall.
2. The cervix or internal os, the outlet of the cavity, is small and frequently made smaller by contraction of the circular muscular fibres or obstructed by flexion.
3. The uterine cavity is a normal cavity and not an abnormal one made by destruction of tissue that requires to be filled up by granulation or new formation of tissue.
4. When uterus is tamponed the material acts as a foreign body, stimulating uterine contractions when there is sufficient vitality of the organ, thus crowding the gauze down into os and cervical canal, plugging the cervix and obstructing and checking drainage.

We all know when gauze is constricted in the cervical canal it cuts off drainage, and yet physicians will pack the gauze tightly into the uterus and cervical canal, then placidly wait for drainage while the septic germs march bravely on, invading new territory and destroying the forces of the patient.

It is argued that the gauze will absorb the discharges, yet almost invariably after curetting the gauze tampon is saturated with blood before the dressing of the patient is completed. In point of fact, the gauze is often saturated with blood as fast as introduced into the uterine cavity, and in a few hours the vaginal portion is coated with mucus, while the uterine portion is forced into the internal os plugging it and cutting off capillary drainage.

In the first and second class of cases referred to above, in which the gauze tampon is used, who has not seen elevation of pulse and temperature due to obstruction by the tampon, relieved as soon as the gauze is removed? In such cases its removal will be followed at once by pent-up discharges, increased drainage and relief of unpleasant symptoms.

The good effects of a tampon used after curetting in the first and second class of cases is not so much due to drainage as to its effect in checking hemorrhage, stimulating uterine contractions, prolonging applications to endometrium and acting as a surgical dressing.

In cases of sterility with small flexed uterus and conical cervix the gauze tampon has its advocates; even then, a few days after the dilatation and curetting a cervical or uterine drainage tube is more beneficial. Not such cervical stems as are generally used, for they obstruct drainage and frequently injure fundus of uterus, but one so constructed as to give free drainage, which can not be affected by contractions of cervix, or become occluded by encroachment of cervical mucous membrane or endometrium, neither should it extend to the fundus producing pressure or irritation. The uterine end should have plenty of large velvet-eyed openings, cervical portion without openings, and as large channel as it is possible to secure, making a very thin shell.

These tubes are made in three sizes of hard rubber by the Davidson Rubber Company, the smaller size being used in these cases.

It acts as a stem, tends to straighten the uterus, stimulates its development and at the same time, gives free drainage.

In these cases and in endometritis with spasmodic contractions of internal os requiring repeated dilatations to secure proper drainage, the use of these tubes obviates the necessity for repeated dilatations and at the same time allows free drainage, thus relieving many cases before tubes and ovaries become involved.

Every gynecologist finds many cases of endometritis, diseased tubes and ovaries due to abortions improperly treated.

Thhse cases can almost invariably be prevented by appropriate treatment at the proper time.

Ninety per cent. of cases of infection after abortion are putrid and not septic infection.

Prolonged absorption from putrid infection will produce pelvic complications, but in these cases if the uterus is emptied early by any practical method, disinfected and drained, they will recover.

The gauze tampon in these cases should only be used to check hemorrhage, prolong disinfection of endometrium and stimulate uterine contractions. These results will be accomplished in from 24 to 48 hours, then the tampon should be removed, after which the uterus will usually drain itself.

If the tampon is left longer it will obstruct drainage, produce constitutional disturbance, increase the risk of pelvic inflammation, set up irritation, increase absorption, with a possibility of infectious material being forced out into the tubes.

DRAINAGE TUBES.

In septic infection the dangers are greatly increased, the course more rapid and disastrous, with pelvic complications more common.

In these tubular drainage is the most essential factor, as it is in cases of infection after labor.

In infection after labor the order is reversed; the greater majority are cases of septic or mixed infection--this difference is due to the fact that but few cases of abortion are handled or examined until infection occurs except where the uterus is completely emptied, hence not infected by the physician, midwife or nurse, but have simple decomposition of uterine contents.

Not so with cases of labor, for the majority are handled or examined at time of labor by physician, midwife or nurse and the uterus more completely emptied, besides the uterus is much larger, more flabby, cervical and vaginal canalas more patulous all of which favor infection, more rapid absorption, with a greater liability to invasion of other organs and tissues.

At full term the involution of the generative organs requires a greater retrograde tissue metamorphosis, increased action of lymphatic and blood vessels.

Nature also establishes at this time a process of elimination and drainage throwing off noxious waste material known as the lochia.

Plug up the cervix and compel the uterus to take up or retain this effete matter and what is the result?

Add to this condition septic infection with consequent germ development, migration, and absorption; then behold the result.

If this is not ruin and devastation sufficient, take a curette and invade the uterine cavity, break down and tear up Nature's barriers and protections against germ invasion, open up as much raw surface as possible adding traumatism so as to increase absorption, and for fear the germs, toxic material, dead leucocytes and debris may chance to get away, stuff the uterus and cervical canal with gauze, and if the patient recovers it may be said, "she recovered in spite of the treatment."

It is useless to talk about removing all the germs and septic material from the uterus by use of the curette in cases of true septic infection.

It is also equally as absurd to expect a gauze tampon to efficiently drain such a uterus.

Even its most ardent advocates advise changing the tampon once in 24 hours if drainage is desired.

I care not how expert the operator may be he can not remove and replace a gauze tampon every day in cases of puerperal septic infection without doing damage to the endometrium and injuring Nature's process of repair, thus producing traumatism and irritation by the manipulation.

Free unobstructive drainage is the most important factor in the treatment of these cases.

Make a free, open channel from the uterine cavity, keeping it clear and Nature will soon establish a current of elimination which not only unloads the endometrium of noxious material but extends its power of elimination outside the uterine body itself by utilizing the blood vessels and lymphatics.

To quote from a former article: "Nature establishes a system of sewerage, the main channel or trunk being the uterine cavity, the cervical and vaginal canals, the blood vessels and lymphats forming the tributaries. Yet we are advised to fill the uterine cavity with gauze, damming up Nature's channel of elimination, preventing the throwing off of effete material from placental site, endometrium, lymphatics and etc., obstructing the egress and retaining the dead leucocytes ladened with germs and toxines annulling phagocytosis producing the very condition we should endeavor to prevent."

Free drainage in these cases can only be attained by use of the drainage tube.

In severe cases, if desired, capillary drainage may be added by carrying a *strip* of gauze up into the uterus *with the drainage tube*, but not a tampon.

This gives ideal drainage and will drain any uterus in any position by changing patient from side to side in bed.

After ten years of practical use of the drainage tube in these cases I am more fully convinced than ever of its utility, not only in the immediate relief of the infection, but in its power to relieve and prevent involvement of the tubes, pelvic peritonitis, pelvic cellulitis, lymphangeitis and even phlebitis.

I will not worry you with report of cases, but will say I have drained several cases with pelvic complications such as salpingitis, pelvic peritonitis, pelvic cellutitis, lymphangeitis, even phlebitis, and, *in some cases* abundant inflammatory exudate which were relieved, the exudate absorbed, and patients are well without sacrificing any of the generative organs.

These remarks apply more particularly to cases of true septic infection and not putrid infection, for the latter will usually get well if the uterus is cleaned out by any method, disinfected and even tamponed.

When once the drainage tube is given an impartial trial in cases of puerperal septic infection coupled with the use of salines, thus draining the alimentary canal, many lives as well as uteri, tubes and ovaries will be saved.

The largest sized tube the cervix will admit should be used and if constructed of hard rubber on the same pattern as the ones presented, the uterus can be irrigated through the tube without its removal, by using a long metal or soft rubber catheter of small size. Such irrigations should be of mild antiseptic solutions or simply boiled water, as harm is frequently done by repeated washing out of uterus with *strong* antiseptic solutions. *Soft* rubber tubing is more satisfactory in draining cases of puerperal infection if properly constructed and placed for its use. See *New England Medical Monthly*, August, 1896.

In conclusion we would say:

1. A uterine tampon is not a true drain and even obstructs drainage in many cases.

2. Capillary drainage is secured by carrying a strip of gauze up into the uterine cavity, not packing it, and then it drains for a few hours only.

3. Gauze can not even act as a capillary drain, when either end or center is constricted, or when coated with mucus.

4. Gauze when saturated with serum, unless it contains an antiseptic, forms a hotbed for germ development.

5. Never tampon the uterus in puerperal septic infection evcept to check hemorrhage.

6. The good effect of a gauze tampon in cases of endometritis and after abortion is not due to drainage, but to its effect as a tampon *i. e.*, checking hemorrhage, stimulating uterine contractions, prolonging medication of the endometrium and acting as a surgical dressing.

7. The uterine drainage tube is the most essential factor in treatment of puerperal infection and the best method of securing *drainage* when demanded in other diseased conditions of the uterus.

8. It will save more lives, relieve and prevent more pelvic complications than any other one factor at our command.

## Figures and Tables

**Figure f1:**